# Book Reviews

**Published:** 1913-04

**Authors:** 


### BOOK REVIEWS

---

42d Annual Report of the Buffalo State Hospital for the year ending September 30, 1912. This is an interesting document, both from the economic, sociologic and medical standpoints. The following extracts seem to us most important from the former, on account of their bearing on problems under active discussion:

Schedule of discharges by percentages for the year ending September 30, 1912:

| | |
|---|---|
| Percentage of recoveries to total admissions | 24.60 |
| Percentage of recoveries to new admissions | 31.71 |
| Percentage of recoveries to average population | 4.60 |
| Percentage of recoveries to number discharged | 26.76 |
| Percentage of recoveries to number discharged, exclusive of deaths, inebriates and transfers | 45.96 |

| | |
|---|---|
| Percentage of recoveries to new admissions, excluding inebriates and drug habitues | 32.04 |
| Percentage of recoveries to all admissions excluding inebriates, drug habitues and transfers | 26.53 |
| Percentage of deaths to average population | 6.42 |
| Percentage of deaths to whole number treated | 5.53 |

Average purchase price and annual per capita cost of staple articles of consumption:

| | Average purchase price | Annual per capita cost |
|---|---|---|
| Fresh meats, per pound | $0.089 | $15.76 |
| Poultry, per pound | .172 | .56 |
| Wheat flour, per barrel | 4.33 | 5.33 |
| Butter, per pound | .268 | 9.515 |
| Cheese, per pound | .139 | .947 |
| Eggs, per dozen | .256 | 4.542 |
| Tea, per pound | .154 | .726 |
| Coffee, per pound | .183 | 1.95 |
| Sugar, per hundred weight | 5.40 | 2.95 |
| Milk (fresh), per gallon | .158 | 8.297 |
| Total for food | | 50.37 |
| Liquor, per gallon | 1.918 | .076 |
| Coal, per ton | 1.826 | 13.00 |

It should be explained that fresh vegetables and fruits are mostly raised on the hospital grounds. The total actual cost per patient is stated at $202.55 (not counting interest on investment, which is, of course, free from taxation.) The total number of physicians is 11, 1:177 patients, and the salaries of the medical officers represent an average expense of $9 a year per patient. Considering that this includes about 125 major operations and that the average daily number of patients confined to bed was 125, it indicates a degree of economy which should be impressed upon the public. Dr. Hurd and his staff are to be congratulated on the efficiency of the management.

---

**Chloride of Lime in Sanitation.** Albert H. Hooker, Technical Director of Hooker Electro-Chemical Co., Niagara Falls, N. Y., published by John Wiley & Sons, N. Y.; 231 pages. No price stated.

While the avowed purpose of this book is to call attention to the efficiency and economy of chloride of lime as a disinfectant, it contains a vast amount of information collected in a careful, scientific study of the subject, including many excursions into allied topics. The statistics of the bulk of sewage, of infant mortality, the bacteriologic tests of water and sewage before and after treatment, the economics of typhoid fever are a few of the details considered. For the sanitary engineer and the physician interested in hygiene, it is invaluable. We especially commend the broadmindedness of the Company in enabling the author to give full scope to his own originality and aptitude for literary research.

---

Proceedings of the Canal Zone Medical Association for the half year, April-September, 1911. Vol. 4, Part 1.

This is an interesting collection of papers, including several on protozoic diseases.

---

Transactions of the Eighteenth Annual Meeting of the American Laryngological Rhinological and Otological Society, 1912, including the meetings of the four geographic sections during the year; 524 pages, besides index; illustrated.

The inclusion of papers on oesophageal diverticulum, the Wassermann reaction, the hypophysis, infections of the meninges, etc., indicates the broad range of this specialty. It is impossible to do justice to the various authors and we will single none out for special mention. The Secretary, Dr. Thomas J. Harris of Philadelphia, is collating and preparing the matter for publication, has performed a large and difficult task with great credit.

---

Diseases of the Stomach and Upper Alimentary Tract. Anthony Bassler, M. D., New York; published by the F. A. Davis Co., Philadelphia; 870 pages, 75 full-page plates, several in color, and over 100 other illustrations; $6.00.

While the author has wisely followed the customary arrangement of such books, one notes a great deal of original matter. Without exaggerating its importance, many excellent X-ray pictures illustrate this method of locating the stomach, foreign bodies, etc. The chemistry of the saliva, gas formation in the stomach, duodenal ulcer, an elaboration of the editor's centrifuge test for indican (approximately quantitative), dietetic standards, are selected at random from the many details included in this work and usually omitted. Unlike the majority of perfunctory writers on gastric analysis, Bassler describes properly the end-point of the test by dimethyl for HC1, but it seems to us that his acceptance of the alizarin test for combined HC1 should be replaced with scepticism. It is questionable, also, whether credit should be given to anyone in particular for the simple test for lactic acid with uncolored solutions of ferric chlorid. The chart to show graphically the relative locational frequency of gastric ulcer is very instructive. One is impressed throughout the work, with the critical and practical standpoint from which the various details are discussed—an attitude only reached by one with a large experience and a keen and logical intellect. It is this quality of authorship which lends value to a book and especially to one entering a field already occupied, or to one in which differences of individual opinion often arise. Dr. Bassler is well known to our readers, both by general reputation and by his recent contribution to the JOURNAL, and they will join with us in extending to him hearty congratulations for his latest literary production.

---

CONTRIBUTION TO THE SURGERY OF BONES, JOINTS AND TENDONS. John B. Murphy, A. M., M. D., LL. D., Chicago. Reprint from the *Journal of the A. M. A.* 71 pages, 167 illustrations.

The very modest title and the paper binding of this reprint are out of all proportion to its merit. Except that the reader is not delayed by a discussion of generally accepted dicta and that actual cases are employed to a considerable degree to develop the argument, this monograph is a very complete presentation of the division of surgery mentioned, rather than a contribution, in the usual sense. It is, however, in another sense, a contribution by this distinguished surgeon to the members of the great national organization which has recently honored him as well as themselves, by an election to the presidency. At the risk of appearing sordid, and solely to impress upon our readers the practical value of supporting professional organization, quite apart from the general advantages of professional solidarity, we take the liberty to point out that, as ordinarily printed, this monograph is equivalent to a 150-page book, fully illustrated, whose regular price would be about $1.50. This is only one of the many valuable returns made by the A. M. A. to its members. Instead of a little over 30,000 members, this organization should have 100,000. Membership is not only a professional duty, but a matter of self-interest of the most practical kind.

---

TEXT BOOK OF OPHTHALMOLOGY, by Dr. Paul Roemer of Griefswald, translated by Dr. M. L. Foster, published by the Rebman Co., 1123 Broadway, N. Y. Vol. 3, pages 573 to 876; 186 illustrations and 13 colored plates for this volume. $2.50 per volume.

We have already spoken in the highest terms of the first two volumes. The present concludes the series, following the same clinical style as made the first two volumes so lucid and practically valuable. The present edition is being rapidly exhausted and it is announced that the next edition will be in a single volume.

---

DENTISTS' DIARY, 1913, published by Lehn & Fink, N. Y.

This is a very convenient account book, suitable for dentists or physicians having an exclusive office practice, the book being too large for the pocket. The page margins contain excerpts from dental literature. The publishers have complimented the editor by several quotations from papers on borderland medico-dental subjects, which are acknowledged with thanks.

---

Steuben Sanitarium Announcement.

This is a neat pamphlet by the Superintendent, Dr. J. E. Walker, and the Assistant Superintendent, Dr. H. R. Carson, giving information as to the equipment, location and purposes of this excellent institution.

---

Governor Sulzer's Message on Public Health With Report of Special Public Health Commission. Transmitted to the Legislature February 19, 1913.

It is a favorable indication that the Governor appointed this commission within ten days of his assumption of office and that his message consists in a brief endorsement of the data and legislation recommended by the Commission. An executive so thoroughly imbued with the importance of prompt and efficient action on this important subject and so modest and generous in giving all credit to the Commission—which served without pay except for clerical assistance—inspires confidence.

---

Muscle Training in the Treatment of Infantile Paralysis. Boston Normal School of Gymnastics, 1905. Reprinted from *The Boston Medical and Surgical Journal*, Vol. CLXVII, No. 17; pp. 567-574; Oct. 24, 1912. Price, 25 cents. W. M. Leonard, Publisher, 101 Tremont Street, Boston, Mass.

The demand for light upon this subject exhausted the file of the Journal in which it was printed and has led Dr. R. W. Lovett and the Medical Journal to re-issue the article in form of a thirty-two page reprint at the nominal price of twenty-five cents. The directions given are explicit and make the reprint not only of great value, but practically the only set of definite directions in the treatment by exercise of conditions following paralysis.

---

An Introduction to Chemical Analysis, for Students of Medicine, Pharmacy and Dentistry, by Elbert W. Rockwood, M. D., Ph. D., head of the Dept. of Chemistry of the University of Iowa; 4th edition; 246 pages; 20 illustrations. P. Blakiston & Co., Philadelphia, $1.50.

While it should be understood that this makes no pretension of being a treatise on chemic theory nor of covering physiologic chemistry, as practised by the clinician, it is an excellent introduction, giving the student facility in both qualitative and quantitative analysis and affording abasis on which more technically medical analysis may be developed.

PRACTICAL MEDICINE SERIES, 1912, VOL. 8. The series is under general editorial charge of Drs. Gustavus P. Head and Charles L. Mix of edited by Dr. George F. Butler; Preventive Medicine by Dr. Henry B. Chicago. This volume includes Materia Medica and Therapeutics, Favill; Climatology by Dr. Norman Bridge of Los Angeles. Published by the Year Book Publishers, Chicago. Price for the volume, $1.50; for the series of ten, $10.00.

As stated for the other volumes of the series, the present having been received out of its turn, this series is an excellent review of periodical literature, all the better because the editors compile from other writings instead of presenting their personal views. Hormonal, Carbon Dioxid Snow, Limonene, Salvarsan and Neosalvarsan, Maps showing distribution of Plague, Deforestration are some of the noteworthy topics discussed.

---

TRANSACTIONS OF THE AMERICAN ASSOCIATION OF GENITO-URINARY SURGEONS, 1912; 315 pages, illustrated, paper cover, published for the Association by F. H. Hitchcock, 105 W. 40 St., N. Y.

The list of members includes only one in our special territory: Dr. Ruggles of Rochester. It is obviously impossible to review adequately, a considerable mass of papers on various subjects and we dislike, in such cases, to single out individual papers. The collection is a most valuable one.

---

MEN, MANNERS AND MEDICINE, by Medicus Peregrinus. Octavo, paper cover; 98 pages; $1.00; published by W. M. Leonard, Boston.

This is an interesting collection of essays and sketches which have appeared in the *Boston Medical and Surgical Journal.* They represent a type of scholarship rare in these pragmatic times, and reminding one somewhat of Oliver Wendell Holmes's prose.

---

NAPOLEON'S CAMPAIGN IN RUSSIA, ANNO 1812, by Dr. A. Rose of New York, published by the author. About 200 pages, with illustrations by O. Merte; $1.50.

This is not exclusively a medical consideration of the campaign, though, naturally, the author has emphasized the professional aspects of it more than an ordinary historian would have done. Dr. Rose is not only a practical physician but known for his scholarly researches, especially in Greek. The study of history furnishes many lessons applicable to daily life and affords more interesting diversion than most fiction. We commend this work to those of similar tastes.

---

PROGRESSIVE MEDICINE, March 1, 1913. Quarterly, $6.00 per annum; published by Lea & Febiger.

The present volume contains Surgery of the Head, Neck and Thorax, by Charles H. Frazier; Infectious Diseases, by John Ruhräh; Diseases of Children, by Floyd M. Crandall; Rhinology and Laryngology, by George B. Wood; Otology, by Arthur B. Duel. The review of recent study of the hypophysis, the cure of 500 cases of Yaws by salvarsan and the study of measles in monkeys are especially interesting. The customary high standard is maintained.

---

TREATMENT OF DISEASE IN CHILDREN, by A. Sutherland, reviewed in March, page 497, is supplied through the American branch of the Oxford University Press, 35 W. 32d St., New York. Our statement that it was "published as above" was correct as we wrote it but was rendered incorrect by a shifting of position by the printer.

---

DISEASES OF WOMEN: Thomas George Stevens, M. D., B. S., F. R. C. S., M. R. C. P., London. University of London Press (Oxford University Press, American Branch, 35 W. 32, N. Y.); 431 pages, 202 illustrations, $5.50.

This is a systematic treatise, well presented and well brought out by the publishers.

---

PSYCHANALYSIS: Its Theories and Practical Application. By A. A. Brill, Ph. B., M. D., Chief of the Neurological Department of the Bronx Hospital and Dispensary; Clinical Assistant in Psychiatry and Neurology at Columbia University Medical School. Octave of 337 pages. Philadelphia and London: W. B. Saunders Company, 1912. Cloth. $3.00 net.

It is rather difficult for one engaged in the more pragmatic medical problems to enter with a sympathetic spirit into the realm of psychanaysis. For example, while a dream may be interpreted in a far-fetched way to indicate some concealed or even unrealized obsession, fear or suspicion or desire of the patient, ordinary dreams seem rather to depend upon external stimuli and, even in their bizarre elaborations of these stimuli, to express ideas derived from reading or conversation rather than subjective tendencies. Possibly there is a distinct cleavage between the dreams of normal and of abnormal persons in this regard. A book of this kind necessarily deals with the lowest forms of human depravity and, for this reason, it does not make pleasant reading, but it must be remembered that the close analysis of the various apropsychoses (if it be permitted to coin a technical term for foul-mindedness) is a necessary step toward their relief. It certainly does not become one who examines intestinal excrement to scorn the man who analyzes mental excrement.

Principles and Practice of Obstetrics. By Joseph B. De Lee, A. M., M. D. Professor of Obstetrics at the Northwestern University Medical School. Large Octavo of 1060 pages, with 913 illustrations, 150 of them in colors. Philadelphia and London: W. B. Saunders Company, 1913. Cloth, $8.00 net; Half Morocco, $9.50.

This is a magnificent work, entering into almost every conceivable detail. There is nothing novel in the arrangement nor in the author's aspirations. Obviously, there can be little that is strictly original in so well established a branch of medical practice. It is the masterly thoroughness of the author, illustrator and publisher, that is impressive.

